# Electroencephalogram-Based Approaches for Driver Drowsiness Detection and Management: A Review

**DOI:** 10.3390/s22031100

**Published:** 2022-01-31

**Authors:** Gang Li, Wan-Young Chung

**Affiliations:** School of Psychology and Neuroscience, University of Glasgow, Glasgow G12 8QB, UK; Gang.Li@glasgow.ac.uk

**Keywords:** drivers’ drowsiness detection, EEG, machine learning, brain stimulation, closed-loop algorithms

## Abstract

Drowsiness is not only a core challenge to safe driving in traditional driving conditions but also a serious obstacle for the wide acceptance of added services of self-driving cars (because drowsiness is, in fact, one of the most representative early-stage symptoms of self-driving carsickness). In view of the importance of detecting drivers’ drowsiness, this paper reviews the algorithms of electroencephalogram (EEG)-based drivers’ drowsiness detection (DDD). To facilitate the review, the EEG-based DDD approaches are organized into a tree structure taxonomy, having two main categories, namely “detection only (open-loop)” and “management (closed-loop)”, both aimed at designing better DDD systems that ensure early detection, reliability and practical utility. To achieve this goal, we addressed seven questions, the answers of which helped in developing an EEG-based DDD system that is superior to the existing ones. A basic assumption in this review article is that although driver drowsiness and carsickness-induced drowsiness are caused by different factors, the brain network that regulates drowsiness is the same.

## 1. Introduction

Driver drowsiness is a major safety concern. For example, the U.S. National Highway Traffic Safety Administration (NHTSA) reports that about 2.5% of the fatal crashes during the period 2005–2015, which resulted in 9142 deaths, were due to drivers’ drowsiness [1]. Although recent statistics show that there is a decreasing trend in drowsy driving-related fatalities, the number of deaths per se is still heartbreaking, with 785 in 2018 and 697 in 2019 [2]. Driverless cars seem to be a ground-breaking and once-and-for-all solution for the driver drowsiness issue. This is especially pertinent given that General Motors, the vehicle tech giant, has already started its self-driving services in San Francisco [3]. Similarly, Baidu, a Chinese tech giant, is promoting its driverless taxi services in Beijing [4]. Driven by these tech giants, the market of driverless cars is expected to reach USD 42 billion by 2025 [5]. However, the aforementioned “once-and-for-all” is actually a misnomer, since self-driving would produce a new problem—self-driving-induced carsickness [6]. Drowsiness is, in fact, one of the most representative early-stage symptoms of carsickness [7]. Self-driving-induced carsickness refers to motion sickness experienced when drivers become passengers, to engage in in-car entertainment activities (e.g., reading and watching movies) or activities related to social productivity (e.g., working). These activities or services can radically improve passenger journeys but, unfortunately, this is only limited to those who do not experience motion sickness. Therefore, early warnings from drivers’ drowsiness detection (DDD) may not only help reduce drivers’ drowsiness being a cause for accidents in traditional driving scenarios, but would also address a serious obstacle in the widespread acceptance of driverless cars. This is particularly imperative in semi-self-driving scenarios, requiring drivers to rapidly take over the control of the car once the self-driving control system is out of its capacity, when facing complicated road situations or in the context of implicit traditional carsickness, originally suppressed by driving behavior (shifted attention) but becomes explicit as the driver becomes a passenger. Thus, DDD approaches presented in this review article are significant references to help design a better DDD system, regardless of conventional driving conditions or emerging self-driving scenarios.

The standard clinical tests for measuring sleepiness are the Multiple Sleep Latency Test and the Maintenance of Wakefulness Test, combined with polysomnography datasets [8]. These measurements are very expensive and cumbersome to perform, because they require at least eight channels, outlined here: four electroencephalograms (EEG), two electrooculograms (EOG), one electromyogram (EMG), and one electrocardiogram (ECG) [8]. For the detection of drivers’ drowsiness, many methods have been proposed, which include the vehicle-based methods (such as the lane departure warning system (LDWS) [9,10] and the steering wheel movement (SWM) system [11,12,13]), video-based methods (such as the detector of the degree (percentage) of eyelid closure over the pupils, over time (PERCLOS) [14,15,16]), and physiological signal-based methods (such as those based on the variability of the ratio of low frequency to high frequency in heart rate [17,18] and EEG (brain waves) [19]). Williamson et al. and Golz et al. reviewed on-road fatigue monitoring technologies in 2005 and 2010, respectively [20,21]. Reviewing the existing sensor-based DDD systems [22], Sahayadhas et al. believed that physiological sensor-based signals offer the most reliable means of detection, because they indicate the true internal state of the driver. Brown et al. reviewed vehicle-based sensor technologies for DDD [23], but their reviews pay little attention to the EEG, although it is a non-invasive physiological means of measuring brain activity and has the closest relationship to drowsiness [8]. Particularly, under similar performing conditions, EEG is reported to perform better than any other physiological signal [24].

**Figure 1:**
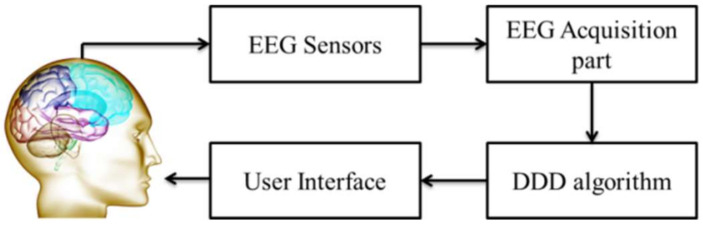
Block diagram of a typical EEG-based DDD system.

Figure 1 shows a generalized block diagram of a typical EEG-based DDD system. In this kind of system, EEG sensors are used to record the noise-contaminated and weak brain bio-potentials. The signals are filtered, amplified and digitized by the EEG acquisition part of the system, until they become clearer and stronger. After that, a pattern recognition techniques-based algorithm will further process these EEG signals for estimating drowsiness levels (open-loop DDD) or for managing drivers’ drowsiness level (closed-loop DDD), via the user interface. Considerable work has been done by some previous authors [25,26,27] on the general aspects of EEG sensors and the acquisition part, but we focus here only on DDD algorithms. Additionally, some unresolved problems as in [25,26,27], such as the EEG montages, including the number, location and type of the EEG channels, are also reviewed.

**Figure 2:**
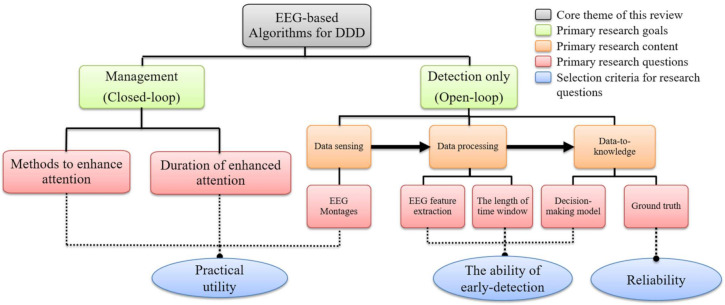
Hierarchical taxonomy for EEG-based DDD algorithms dealing with seven primary research questions and corresponding selection criteria for these questions.

We propose a taxonomy (Figure 2) to address the open-loop and closed-loop problems of EEG-based DDD algorithms. The taxonomy of open-loop problems is based on the standard pattern recognition processing chain, which is introduced in [28]. Availing internet searches, using the tag words “drivers’ drowsiness” or “drowsiness”, on IEEE Xplore, ScienceDirect, and SpringerLink, addressed seven fundamental questions relating to the development of better and more reliable DDD algorithms.

(1) Which is the most suitable EEG montage for DDD?
(2) Which is the most suitable time window length for extracting EEG feature?
(3) Which is the best EEG feature for DDD?
(4) Which is the best decision-making (DM) model for DDD?
(5) Which is the most reliable ground truth for DDD?
(6) Which methods can be used to enhance the driver’s attention?
(7) Which attention-enhancing methods has the longest duration?

The criteria followed in selecting these seven questions was based on the three following aspects:

(1) Practical utility (addressed by questions (1, 6 and 7))
(2) Early-detection of driver drowsiness (addressed by questions (2, 3 and 4))
(3) Reliability (addressed by question (5))

## 2. Taxonomy

The open-loop and closed-loop problems relating to the field of EEG-based DDD algorithms can be organized into a taxonomy, as shown in Figure 2. The components of this taxonomy are briefly discussed below:

### 2.1. Data Sensing

Our focus here is on the EEG montages (e.g., the number, location and type of the EEG channel) to be used in DDD. EEG montages have a close relationship with the difficulty in wearing the headset and to power consumption. Unlike the well-documented montages in a clinical sleep-scoring system [29], there is no standard montage document for EEG-based DDD. Therefore, to design a low-power and simple-to-use algorithm, the primary requirement is to identify the right type of EEG montages.

### 2.2. Data Processing

Another issue we focused on was the feature extraction method, which is used to generate a set of features from raw EEG signals. The other related issue is the length of time window, because it determines the frequency of EEG features generation, which is important in the ability of early detection.

### 2.3. Data-to-Knowledge

This research content involves an understanding of the generated features. However, unlike the application-dependent problems (e.g., data sensing and processing), DM methods are more general, i.e., DM algorithms can be used for a wide range of features. Therefore, instead of focusing on the mathematical model, we focused on detection accuracy and the type of DM model (i.e., two-class classification, multi-class classification or probability estimation). The type of the DM model can indicate the ability of early detection of the DDD algorithm. For example, a two-class classification model only determines whether the driver is drowsy or not, it cannot further evaluate the driver’s degree of drowsiness; a multi-class classifier can be used to estimate the severity of drowsiness so that it can provide the drivers with an early warning of drowsiness. The other focus here is the ground truth. Ground truth is used to label truly alert and drowsy events, which is very important for developing the supervised machine learning based DM model. The reliability of ground truth determines the reliability of the developed DM model.

### 2.4. Methods to Enhance Attention

Our focus here was to review the closed-loop DDD algorithms, which includes methods to enhance drivers’ attention. This kind of algorithm not only detects drowsiness, but also tries to arouse the driver, via effective feedback, which is a real-world DDD solution having strong practical utility.

### 2.5. Duration of Enhanced Attention

As we know, the best solution for drowsiness is rest. Therefore, the acceptable duration of enhanced attention by arousing feedback must allow drivers to have enough time to drive to the nearest parking area (PA) or service area (SA). Hence, our focus here was to further investigate the methods mentioned in (4) and find out the longest duration of enhanced attention after arousing feedback. This is the critical factor in developing a successful closed-loop DDD algorithm.

## 3. Open-Loop Problems

### 3.1. Data Sensing

#### 3.1.1. Number of EEG Channels

The number of EEG channels used in DDDs varies widely, as shown in Table 1. However, most (58%, 32 out of 55) use single-channel or two-channel EEG signals, while only a few use more than five-channel EEG signals (24%, 13 out of 55).

#### 3.1.2. Location of EEG Channels

**Figure 3:**
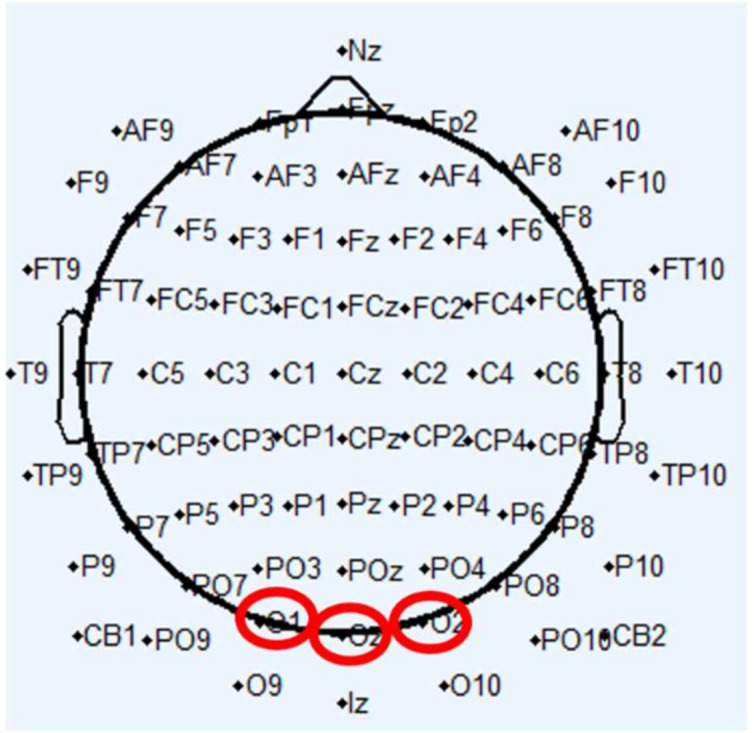
The widely used EEG electrode locations. Totally, 81 EEG channel locations are presented, in which the red circles refer to commonly used occipital region.

Figure 3 shows the EEG 10–20 international system, which is adapted from EEGLAB Toolbox (ver. 7.1.3.13b), using the built-in official location file (Standard-10-20-Cap81.locs) [30].

Many studies (42%, 8 out of 19) have used the occipital region (e.g., O1, O2, Oz) for single-channel EEG. For two-channel or multi-channel EEG, there is no obvious preference for channel location. For example, the locations can cover all EEG channel locations, including the occipital, parietal, temporal, central and frontal regions. However, the occipital region is the most favored, as at least one occipital location is combined with other locations for the majority of two-channel or multi-channel studies (69%, 18 out of 26).

#### 3.1.3. Type of EEG Channels

For single-channel EEG, the majority (53%, 10 out of 19) used bipolar channel (e.g., O1&O2), whereas for two-channel EEG, the majority (55%, 6 out of 11) used unipolar channel (e.g., C3, P3). For multi-channel EEG, they all used unipolar channels.

#### 3.1.4. Discussion

In real-world DDD application, the EEG headset is usually wireless and powered by a battery. As we know, the more sensing channels used, the more power is consumed. Therefore, single channel should be the most favored number of channels for an EEG-based DDD algorithm.

Regarding the channel position, Lin et al. pointed out that the EEG changes at occipital and central regions have a strong correlation to drivers’ drowsiness [31]. The most popular EEG channel for DDD is located in the occipital region; the occipital EEG is highly correlated to the driver’s level of vigilance [32]. The physiological reasoning behind the “success” of the occipital region is associated with the visual cortex. The visual cortex, which is responsible for processing visual information, is located in the occipital region of the brain. When the drivers transition from alert status to drowsy status, the duration and frequency of their sideways glances increase. Their eyes become glazed and the degree of their eye opening decreases, to the extent of becoming an almost complete closure, and the duration of their eye closure increases [33]. These drowsiness-related eye movements blur the visual field and reduce visual input, causing EEG changes (e.g., the increased *α* wave) [34], where [34] is the work from our group.

One challenge for occipital EEG is that the occipital region is hairy. In real-world applications that involve the usage of dry electrodes, the bodily movements of the drivers, such as rubbing the eyes and face, yawning, and moving restlessly on the chair, would influence EEG signal quality and result in unreliable measurements [87], where [87] is the work from our group. This problem can be solved by integrating the motion sensor into the headset and using it together with EEG to detect drivers’ drowsiness facilitates enrichment of the contextual information lost by EEG [88], where [88] is the work from our group.

**Figure 4:**
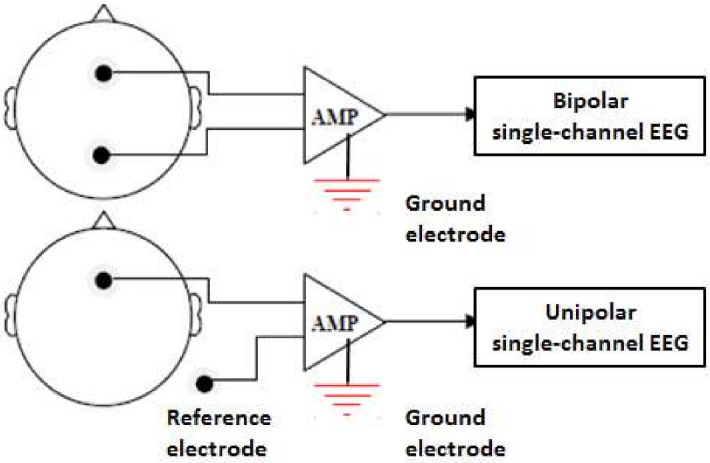
The type of EEG channels. Bipolar type (**top**) and unipolar type or monopolar (**bottom**).

The type of EEG channels used may consist of either a unipolar or a bipolar channel (Figure 4). In the case of a unipolar channel, each electrode records the potential difference, which is compared to that of a neutral electrode, connected to an ear lobe or mastoid. Bipolar measurements show the potential difference between two paired electrodes. Currently, the majority of DDD studies focus on the EEG difference between alert and drowsy statuses, and few of them reported the hemisphere superiority phenomenon. Therefore, the bipolar channel may provide sufficient information for EEG-based DDD. However, for EEG-based emotion recognition research, for example, a unipolar channel is more suitable because it is necessary to compare the EEG changes between the right and left hemispheres [89].

### 3.2. Data Processing

A wide range of EEG features relevant to DDD can be grouped into the following six categories: pure time domain-based features, fast Fourier transform (FFT)-based features, higher-order statistics (HOS)-based features, wavelet-based features, other time-frequency-based features, and hybrid features. Among these, the most popular features are FFT-based features (71%); the most commonly used length of time windows to extract these features are 1 min (25%), 30 s (16%), 1 s (14%) and 2 s (13%).

#### 3.2.1. Time Domain Features

Barring the fractal dimension, only a few approaches use time domain features alone to identify the characteristics of EEG time series that vary between the alert and drowsy states. Usually, time domain features are used in combination with FFT- or wavelet-based features.

##### Fractal Dimension (FD)

A frequently used measure of complexity is the fractal dimension (FD). Some studies use the FD alone to detect drowsiness. For example, Michail et al. [39] computed the FD values of an EEG signal, using Higuchi’s method [90]. Their results show that the FD values tended to decrease as the subjects became drowsy. Tantisatirapong et al. [47] employed two algorithms to calculate the FD (variance fractal dimension (VFD) and detrended fluctuation analysis (DFA)). They found that the VFD method is superior to other methods for analyzing alertness and drowsiness patterns.

##### Other Time Domain Features

Table 2 summarizes the other time domain features that are used in EEG-based DDD, with the following notation: $x_{k}\in \left\{x_{1}\ldots x_{N}\right\}$. The notation denotes the *k*th EEG time samples *x*, in which *N* is the number of *x*. The mean, first derivative, second derivative, variance, covariance matrix, window length and sampling rate of *x* are respectively denoted by μ, $\dot{x}$, $\ddot{x}$, *var*, *S*, *t* and *f_s_*.

#### 3.2.2. FFT-Based Features

Assuming that the EEG signal is stationary, FFT can be applied to extract the EEG features. FFT-based features are generally FFT power features from different EEG frequency bands, including the Delta band (*δ*: 0 to 4 Hz), Theta band (*θ*: 4 to 7 Hz), Alpha band (*α*: 8 to 12 Hz), Beta band (*β*: 12 to 30 Hz) and Gamma band (*γ*: 30 to >100 Hz). These band power features can be directly fed to a DM model to detect driver’s drowsiness. Alternatively, the relative band power, various band power equations, or the log band power features can also be used. Also, a few approaches combine FFT and time- or other frequency-domain feature extraction methods to generate novel EEG features for DDD. We call this method as “FFT+”. Table 3 summarizes these FFT-based features.

##### Pure Band Power (PBP)

Among the five EEG frequency bands, *θ*, *α*, and *β* bands are more popular than *δ* and *γ* bands for detecting drowsiness. The physiological reasons for this are as follows [16,25,40]: (1) when the driver transitions from the alert to the sleepy state, *β* power decreases; (2) when the driver is in a relaxed state, with his or her eyes closed, *α* power becomes abundant; (3) when the driver enters the standard sleep state, *β* and *α* powers gradually diminish, giving rise to *θ* power. Numerous studies have been carried out using PBP as an EEG feature for DDD. For example, Chae et al. [58], Kim et al. [59] and Yang et al. [91] employed *α* power as the single feature, while Sun et al. [79] used *α* and *β* powers, and Lin et al. [62] and Wang et al. [67] used *θ* and *α* powers as double features. Park et al. employed the following three features: *θ*, *α*, and *β* powers [49]. Cao et al. [35] and King et al. [44] used the following four features: *δ*, *θ*, *α*, and *β* powers. Liu et al. used all five EEG band powers, from *δ* to *γ* [92].

##### PBE

There are several PBEs in EEG-based DDD (see Table 3). These equations consist of different pure/weighted band powers, and their outputs are used as input features for DDD.

##### RBP

“Relative band power” implies that the authors used EEG power percentages, instead of the absolute EEG power values, as the input features for DDD. The RBP is calculated by dividing the FFT power of one EEG band by the aggregate FFT power of all the EEG bands employed, as shown in Table 3. Picot et al. used the RBP of *α* and *β* [61], Zhang et al. and Awais et al., an RBP of *δ* to *β* [66,82] and Papadelis et al., an RBP of all five EEG band powers [19].

##### Single-Hz Band Power (SHBP) and LBP

Unlike the five commonly used frequency band powers (*δ* to *γ*), the frequency band power can also be computed in small equal-sized bins. For example, Yu et al. used two-band power features in a 1 Hz resolution bin, which have averages of 1 to 4 Hz power, and 9 to 11 Hz power [72]. Putilov et al. employed SHBP values in the frequency range of 1 to 16 Hz, as 16 features [71]. The normalizing of the EEG band power to the logarithmic scale was first reported in [93,94]. The EEG band power correlates with the wake–sleep transition more linearly in the logarithmic scale than in the linear scale. Lin et al. confirmed this phenomenon by using the five best single-Hz width LBPs that are near the *α* band (e.g., 8 to 12 Hz or 10 to 14 Hz) [26]. Also, Putilov et al. proposed a novel drowsiness indicator, which showed the difference between the log-transformed *α* power and *θ* power [71]. Lin et al. employed SHBP values in the frequency range of 1 to 30 Hz as features [77].

##### FFT+

Reddy et al. used the SE of the power spectrum, based on the steady state visually evoked potentials (SSVEP), to detect drivers’ drowsiness [36]. For an alert subject, the power spectrum is narrow and has the peak frequency at the flicker rate of the steady pattern, while for an inattentive subject, this peak is no longer prominent. Also, the SSVEP was more evenly spread over the spectrum, and the authors calculated the SE of the power spectrum of SSVEP. If the subject becomes drowsy, the SE will increase, relative to that of the alert state. In this study, the steady state stimulus (LED) is placed on the car’s rear view mirror.

Lin et al. presented a technique for which they combined the MD to detect driver’s drowsiness [43]. They employed the MD of *θ* (termed MDT) and the *α* power spectrum (termed MDA) as a measure of drowsiness, by analyzing the variations in MD over time. Specifically, if the driver is alert, his or her EEG spectra in the *θ* and *α* rhythms will follow a multivariate normal distribution, which can be characterized in the alert models. Next, the deviation of the driver’s state will be assessed continuously from the alert model by using MD. If the driver remains alert, his or her EEG spectra in *θ* and *α* rhythms should match with those of the alert model. Otherwise, if the driver becomes drowsy, then his or her EEG spectra will deviate from those of the respective model and, hence, MD will increase.

Picot et al. combined RBP and MCT to detect drivers’ drowsiness [61]. They employed the MCT value of relative *α* and *β* powers as a new drowsiness indicator (see the last two rows of Table 2). The calculation of MCT requires a fixed reference window (60 s length) and a moving window (20 s length). The RBP features of the fixed window are calculated at the beginning of the driving session for each driver, when the driver should be fully awake. The RBP features of the moving window are calculated as time goes by and compared with (subtracted by) the values of the fixed window every 10 s. The advantage of this method is that it is so normalized that the same detection threshold can be used for different drivers. Hence, they believe that the method is robust to inter- and intrapersonal differences, as also to age-related differences that may influence drowsiness detection.

Hal et al. divided *α* and *β* bands into the following four sub-bands: low *α* (7.5 to 9.25 Hz), high *α* (10 to 11.75 Hz), low *β* (13 to 16.75 Hz), and high *β* (18 to 29.75 Hz). Then, eight features, comprising the mean and STD of each sub-band, were extracted [83]. Hu et al. employed the dominant frequency (DF), average power of the dominant peak (APDP), center of gravity frequency (CGF), frequency variability (FV), and mean power frequency (MPF) from *δ* band to *β* band as features [69]. Reports of using the MPF feature can also be found in [57,84]. Aboalayon et al. proposed a method that uses integrated EEG (IEEG), SE, and STD, extracted from all the five EEG bands (*δ* to *γ*) as features [75].

A few approaches focus only on the characteristics of *α* band. For example, Simon et al. proposed an algorithm to extract *α* spindle, which is a short narrow band, bursting in *α* band [53]. They evaluated various *α* spindle-related features, including the spindle rate, duration, spectral amplitude, and peak frequency. All the spindle features outperformed the pure *α* power, thus establishing the spindle rate as the best feature. Pritchett et al. explored *α* rhythm by analyzing its burst duration, mean amplitude, relative amplitude, amplitude variance, wave duration variance, wave similarity, and slope smoothness [50]. Kalauzi et al. proposed a method for analyzing the phase information of *α* rhythm [63]. They treat *α* rhythm as a stable frequency with variable amplitude signals and one carrier frequency (CF), allowing for the calculation of *α* CF phase shifts (CFPS) and the development of CF phase potentials (CFPP). They find that the greatest changes in *α* CFPS, CFPP, and phase locking occur in subjects’ frontal regions, while transitioning from the awake state to the drowsiness state.

#### 3.2.3. HOS-Based Features

Unlike the Fourier power spectrum (2nd-order statistics), the bispectrum (*Bis*) consists of 3rd-order statistics, which preserve the Fourier phase information. The *Bis* can be estimated by measuring the 3rd-order cumulant of the EEG samples and then taking a 2D-Fourier transform (as shown in Equation (1)).

(1) $$Bis^{x}\left(\omega_{1},\omega_{2}\right)=\overset{\tau_{1}=+\infty }{\underset{\tau_{1}=-\infty }{\sum }}\overset{\tau_{2}=+\infty }{\underset{\tau_{2}=-\infty }{\sum }}C^{x}\left(\tau_{1},\tau_{2}\right)e^{-j\left(\tau_{1}\omega_{1}+\tau_{2}\omega_{2}\right)}$$

Abeyratne et al. proposed a *Bis*-based novel feature for detecting drowsiness [8], using a single-channel EEG. They employed a single-dimensional slice of the *Bis*, defined as $Bis^{x}\left(\omega ,\phi \omega +\rho \right)$, to estimate the bispectrogram time series (BTS) and find that the amplitude of BTS at *f* = 20 Hz (*ξ*_20_) offers an ability to detect micro-sleep events (the drowsy events before standard sleep Stage I). This slice is inclined to the $\omega_{1}$-axis at an angle tan^−1^*ϕ* and has shifted from the origin, along $\omega_{2}$-axis by $\rho \left(-\pi <\rho <\pi \right)$.

Based on this finding, the sleepiness index (SI) is further defined to measure drowsiness, as shown in Equation (2), where *A*() denotes the amplitude of BTS, *S*_0_ the amplitude threshold for detecting micro-sleep, *Time*() the time maintained, and *Time_total_* the total time.

(2) $$SI=\frac{Time(A\left(\xi_{20}\right)>S_{0})}{Time_{total}}$$

*SI* has a range of 0 to 1 and is very similar to the PERCLOS video-based feature. PERCLOS assesses drowsiness by measuring slow eyelid closure and estimating the proportion of time during which the eyes remain 80% closed over a 1-min interval (high sensitivity), 3-min interval (medium sensitivity) or 5-min interval (low sensitivity) [15]. The formula indicating high sensitivity is given by Equation (3), where ECD is the degree of eye closure.

(3) $$PERCLOS=\frac{Time\left(ECD\geq 80\%\right)}{1\;\min}$$

#### 3.2.4. Wavelet-Based Features

Assuming that the EEG signal is non-stationary, wavelet transform can be applied to extract the EEG features. In EEG-based DDD, the wavelet-based features are generated in the following two ways: discrete wavelet transform (DWT) and wavelet packet transform (WPT). The DWT decomposes the given signal into a set of approximate (*Ai*) and detailed (*Di*) coefficients of level *i* (*i* = 1, …, *n*). The frequency range of each level is calculated as shown in Equation (4) [17], where *n* is the notation of the index of the level and *f_s_* is the sampling rate for the signal.

(4) $$Frequency\_range=\left(\frac{1}{2^{n+1}}\sim \frac{1}{2^{n}}\right)\times f_{s}$$

The regular wavelet decomposition method may not always yield the best results in recognizing patterns [95]. Therefore, a WPT decomposition can be used. WPT decomposes not only the approximate coefficients, but also the detailed coefficients. Therefore, the information (high frequency) which is lost in DWT can be retrieved by using WPT. This explains why WPT is favored in EEG-based DDD.

##### Band Power

Using Equation (15), the DWT or WPT method can also categorize the EEG signals into *δ* to *γ* frequency bands. For example, Akin et al. decomposed the EEG signal into three levels by using DWT and also extracted PBPs in *δ* to *β* bands as features [40]. Gupta et al. used DWT to decompose the EEG signal into four levels and employ PBP in a band (no specific information) and BPE 9 as features [45]. Lee et al. divided the EEG signal into six levels by employing WPT and using PBP in *δ* to *β* band and BPE 1, 3, 4 and 7 as features [96].

##### Wavelet+

We used Wavelet+, just as FFT+ (see Section 3.2.2), to represent the feature extraction methods that combine wavelet transform and time or other frequency domain feature extraction methods for generating novel EEG features. For example, Gupta et al. extracted SE and RE from each band (*δ* to *β*) as features [45]. Lee et al. employed the CGF and FV from each band (*δ* to *β*) as features, and then a mutual information (MI) technique to select the most descriptive features for further classification [96]. Khushaba et al. decomposed the EEG signal via WPT, using the symmlet 5 (“sym5”) wavelet and constructed features by using the normalized logarithmic energy of the wavelet packet coefficients [24]. In feature selection, both Khushaba et al. and Daphne et al. used a fuzzy mutual information-based method for WPT analysis (FMIWPT) [24,64]. Tsai et al. introduced feature extraction via DWT, using the Daubechies 2 (“db2”) wavelet, extracting NZC and IEEG from each band (*θ* to *β*). In this case, a total of 36 features were extracted for classification [42]. Gurudath et al. divided the EEG signal into five levels, using “db3”-based DWT to extract the mean, median, variance, STD, and mode of the bands (*δ* to *γ*) as features [81]. Murugappan et al. decomposed the EEG signals into four bands (*δ* to *β*), using WPT, after which they used FFT to extract the band power and spectral centroid (SC) from the above frequency bands. In this work, four wavelet functions (“db4”, “db8”, “sym8”, and “coif5”) were used, of which “db4”was found to be the best one, in terms of the band power feature [73].

#### 3.2.5. Other Time-Frequency-Based Features

Yoshida et al. proposed a novel feature, called Instantaneous Equivalent Bandwidths (IEBW), based on positive time-frequency distributions (PTFD) [47,60]. The method using the IEBW involves the tracking of the bandwidth changes of random signals. They applied IEBW for EEG analysis and found that the EEG signal for the period when the subjects were trying to remain in the awake state, by fending off sleepiness, has a wider bandwidth than that of the signal for the period of the normal onset of sleep.

#### 3.2.6. Hybrid Features

A comparison of the FFT+ and Wavelet+ features reveals that there is nothing new in the generation of hybrid features. The authors employ different feature extraction methods and feed these extracted features directly to the classifier. For example, Khushaba et al. proposed the time domain autoregressive (TDAR) features, which combine time-domain features and FFT features [70]. These features include the NZC of the EEG raw data (one feature), Hjorth parameters (three features), RMS (one feature), ARMC (ten features), the spectral moments (four features), waveform length (one feature), and Barlow parameters (three features). Garcés et al. [46,80] first used FFT to extract features, such as the central frequency (CenF), peak frequency (PF), ratio H/L (RH/L), the first and third quartile frequency (Q1F and Q3F), spectral STD, the maximum frequency (MF), interquartile range (IR), asymmetry coefficient (AC) and kurtosis coefficient (KC). They then employed the “db2”-based DWT to extract ZC and IEEG from each band (*θ* to *β*). In addition, they also took into consideration the time domain features, including the max, min, and STD values of the EEG signals.

#### 3.2.7. Discussion

Most of the EEG features for DDD are based on the traditional EEG frequency bands, regardless of whether they are generated by FFT or Wavelet methods. Only a few studies extracted novel features. For example, a new feature, *ξ*20, was generated by bispectrum analysis with a 30-sec time window [8]. Based on the “gold standard” of scoring sleep, this feature can track the gradual development of drowsiness (micro-sleep events) until standard sleep Stage I. Another novel feature, IEBW, was generated by PTFD analysis with a 10-sec time window [37]. This feature can differentiate between the wakefulness maintenance state against sleepiness and the normal onset of sleep. These two studies quantitatively proved the unreliability of those studies which directly categorize drivers’ drowsiness as the normal onset of sleep (the first 30 s of sleep Stage I), such as in [46,66,72,75,80]. However, the two features require intensive computation and, thus, their real-time performance needs to be further evaluated.

The length of the time window for feature extraction is directly related to the timeliness function of the DDD system. For example, heart rate variability (HRV) is the widely-used drowsiness indicator for ECG and photoplethysmogram signals [51]. The lengths of minimum and regular time windows for HRV analysis are 3 min and 5 min, respectively [97]; in contrast to this, we find that 1 min is the most favored length of time window for EEG-based DDD methods. From the viewpoint of timeliness, the EEG signal is more suitable for DDD applications. The physiological reason behind the shorter time window for EEG analysis is its direct relationship to drowsiness. Critically, for EEG per se, the novel feature with the shortest time window is the best; that is, PTFD with a 10-sec time window.

### 3.3. Data-To-Knowledge

We next consider ground truth and DM models.

#### 3.3.1. Ground Truth

Ground truth is used to label truly alert and drowsy events, which is important for developing the DM model for DDD. Table 4 lists the ground truths used in previous studies; in Table 4, KDS, KSS, ESS and RK stand for Karolinska drowsiness scoring, Karolinska sleepiness scale, Epworth sleeping scale and Rechtschaffen and Kales. The ground truths 1~3, 11 and 22 are perhaps effective in recognizing general purpose, inattentive driving patterns, including drunk driving, stressful driving and distracted driving, but in comparison to ground truths 10, 14, 15 and 17, they may not be so suitable for DDD. Ground truth 8 is too dangerous to implement. Ground truths 9 and 19 are well-known standards for scoring the sleep stage, which is not directly related to drowsiness. Other ground truths listed in Table 4 are self-assessment-based, which are not reliable. To the authors’ best knowledge, among the ground truths listed in Table 4, PERCLOS is the only method verified for real life applications [14,15,23]. In addition, [23] recommends a novel ground truth, termed PERCLOS+, which combines PERCLOS and SWM for early detection of drivers’ drowsiness.

#### 3.3.2. DM Models

##### Pure Threshold-Based Model

Threshold-based methods, though not ideal for detection tasks [21], have been studied by many because of their simplicity. Table 5 shows the detection accuracies obtained by using threshold-based models. The accuracy (Acc), specificity (Spec) and sensitivity (Sens) listed in Table 5 were calculated using Equation (16), where TP is true positive, TN is true negative, FP is false positive, and FN is false negative. Therefore, Sens indicates how well this classifier can recognize a driver being in the drowsy state, and Spec, a driver being in the alert state. Equation (5) is also applied to Table 4.

$$Acc=\frac{TP+TN}{TP+FN+FP+FN}\times 100\%$$

$$Sens=\frac{TP}{TP+FN}\times 100\%$$

(5) $$Spec=\frac{TN}{TN+FP}\times 100\%$$

##### Binary Classification Model

Similar to the threshold-based methods, binary classification methods categorize the extracted features into the following two classes only: alert and drowsy. However, the binary classification results may be better than those obtained with threshold-based methods, because machine learning techniques can make the decision plane more flexible and match more complicated cases. Table 6 lists the detection accuracies obtained by previous studies, using binary classifiers, where RBF, SVM, ANN, FI and LDA stand, respectively, for radial basis function, support vector machine, artificial neural network, fuzzy interference and linear discriminant analysis.

##### Multi-Class Classification Model

The multi-class classification system can estimate drowsiness levels and provide drivers with a much earlier warning than that possible with the threshold-based or binary classification system. Table 7 lists the detection accuracies obtained by previous studies, using the multi-class classifier, where FNPA stands for fuzzy neighborhood preserving analysis.

##### Regression Model

Unlike the threshold-based and classifier-based methods, which merely estimate discrete labels, the regression model can estimate a continuous dependent variable (e.g., the driving performance indicator) by using one or more independent variable (e.g., extracted EEG features) and can, thus, increase the detection resolution. The commonly used methods for estimating the performance of the proposed regression model are the squared correlation coefficient, denoted by *r*^2^, and the root mean square error (RMSE). Table 8 lists the detection accuracies that can be attained by using the regression models, where SVR, MLR and SONFIN stand, respectively, for receiver support vector regression, multiple linear regression and self-organizing neural fuzzy inference network.

##### Probabilistic Model

Several researchers employed a probabilistic model to predict driver drowsiness. For example, the dynamic Bayesian theory-based posterior probabilistic models (PPM) are proposed with PBP features by [91], and with FFT+ features by [57,84]. However, no mention was made of detection accuracies. Let “+1” and “−1” represent, respectively, drowsy driving class and alert driving class. The posterior class probabilities, i.e., P(class = +1|$\vec{x}$) can represent the probability of drowsy driving. Therefore, the primary advantage of the PPM is that it enables the estimation of the relative severity of drivers’ drowsiness. In this case, the probabilistic mode-based system would have the potential to combat drivers’ drowsiness at an early stage, when feedback might be most effective. However, very few works are included in this aspect. Li et al. and Zheng et al. proposed a SVM-based and continuous conditional neural fields (CCNF)-based PPM in 2015 and 2017, respectively [86,87]. Based on the same ground truth as PERCLOS, they obtained 83.78% and 88% accuracy for early-detection of driver drowsiness, using RBP-based and FFT+-based features, respectively.

##### Transfer Model

The transfer model is a new development of the aforementioned traditional DMs. The fundamental difference between the transfer model and traditional DMs is that the transfer model allows the feature space and distribution in training and testing data to be different [98]. Therefore, the generalizability of the developed DMs could be improved. Thus, considering the nature of the moment-to-moment change and individual differences of EEG, transfer model-enhanced DMs are necessary for the development of EEG-based DDD. However, few works focus on this topic. In 2015, Lin et al. developed a transfer learning-enhanced regression model for EEG-based DDD. By using SHBP features and ground truth 1, this model obtained 70% accuracy, which is higher than the traditional regression model-based 65% accuracy [98].

#### 3.3.3. Discussion

Timeliness is a big challenge to DDD. To address this problem, one needs to adopt not only a shorter data processing time window, but also a smart DM model that ensures timely estimation of drivers’ drowsiness. Our review of the existing DM models of EEG-based DDD shows that they can be classified into the following five categories: pure threshold-based models, binary classification models, multi-class classification models, regression models and probabilistic models. Of these, the multi-class classification model, which classifies drivers’ drowsiness into several levels, is obviously better than threshold-based or binary classification models. However, it is still inferior to the probabilistic model (or regression models), which transforms the drowsiness level to any value of (0,1) (or a continuous variable), instead of discrete labels. However, from the perspective of real-life applications, the higher the resolution of the device used for DDD, the more the computations (thus, more power consumption) required.

Considering all the models, we found that their best detection rates, except that of the probabilistic and transfer model, could exceed 90%. However, the models and the extracted features, which could achieve the best detection rate for each category, do not show any consistency, as evidenced by the following results: BPE 8 for threshold category (Acc = 90.4%), the combination of SHBP and BEP 5 and RBF-SVM model for binary classification category (Acc = 97.48%), Wavelet+ and LDA model of multi-class classification category (Acc = 97%) and LBP and RBF-SVR model for regression category (Acc = 93.2%). In addition, from the perspective of cross-category, we identified four studies for ground truth 1 [44,63,65,78] and four for ground truth 9 [73,77,81,84]. We found that the best overall accuracy for ground truth 1 was obtained using an RBF-SVR model, using log-transformed SHPB (1~30 Hz) features (Acc = 93.2%), and that for ground truth 9 by an RBF-SVM model, using SHPB (1~27 Hz); and BPE 5 features (Acc = 97.48%). While great performance is achieved by ground truth 9, this method categorized driver drowsiness as sleep onset, even when a fatal traffic accident could have already occurred.

## 4. Closed-Loop Problems

### 4.1. Methods for Vigilance Enhancement

The methods for vigilance enhancement to enhance drivers’ attention include visual, vibrational, auditory and non-invasive electronic current-based brain stimulation techniques. Specifically, for visual feedback, the display of an alert icon is proposed [99]. However, Belz et al. [100] found that drivers are less sensitive to visual feedback, because they have to pay continuous attention to road conditions and the dashboard. For vibrational feedback, a built-in vibration sensor in a wristband device (e.g., smartwatch) is proposed [99], but not experimentally validated. For auditory feedback, Lin et al. proposed a 1750 Hz tone-burst, after comparing it with 500 Hz and 3000 Hz tone-burst [38]. They show, in a simulator, that when the drivers are drowsy, the 1750 Hz auditory feedback could decrease *θ* and *α* power in the occipital region [101]. Brain stimulation-based methods, which increase the drivers’ attention and concentration by stimulating the brain with small amounts of direct electronic current [102,103,104,105,106], are worth considering in this regard. The electrodes could be placed on F3 and F4 or non-hairy forehead Fp1 and Fp2 (in accordance with EEG 10-20 International System [104,105,106]). It is important to note that [106] did stimulation at the stage of early drowsiness, when the intervention might be the most effective and necessary.

### 4.2. Duration of Enhanced Attention

Lin et al. mentioned that the 1750 Hz auditory feedback could maintain driver’s attention for up to 40 s [67], a time lag that is enough to stop the vehicle safely, whereas with alternating current-based methods, such as cranial electrical stimulation, the attention maintenance could reach 4 h [102]. It is very important to note that the maximum duration of enhanced attention is reported by [103], in which it could reach 6 h with direct current-based methods, such as transcranial direct current stimulation (tDCS). According to the standard space of each freeway rest area (about 20 km) [107], and assuming the average car speed is 80 km/h on the freeway, we can easily calculate that an acceptable duration of enhanced attention would be 25 min. From this point of view, auditory feedback [67] is not an acceptable solution for closed-loop DDD on the freeway, while tDCS approaches [103,106] can basically benefit drowsy drivers on the freeway. Table 9 shows the details about the methods to enhance attention.

### 4.3. Discussion

Besides the long-lasting effect, another advantage of using brain stimulation methods is that they can work together with EEG neurofeedback approaches innately to form a real-time closed-loop solution for driver drowsiness management. However, like the distracted driving caused by visual, vibration or auditory methods, or the caffeine addiction caused by energy drinks to the drivers, brain stimulation methods have their own side effects, such as tingling, itching and burning sensations. More importantly, the specific stimulation area is still an open question. Based on the existing studies, frontal areas seem to be the commonly used areas. This is most likely because the brain’s frontal lobe is in charge of cognitive control abilities [108], whose enhancement can indirectly suppress the development of drowsiness or reduce the awareness of drowsiness. Actually, apart from the stimulation area, the type of electric current is also an open question. This is especially pertinent given that a more recent study adopted transcranial alternating current stimulation (tACS) to improve participants’ driving performance in a driving video game [109,110]. Like tDCS, tACS is a non-invasive brain stimulation technique that delivers weak electric currents through the scalp, on the order of 1–2 mA. However, as its name implies, tACS delivers sinusoidal alternating current, rather than direct current. This brings another open question, that is, which frequency of the alternating current should be used. Although 6 Hz-theta frequency was used in [109,110], a more comprehensive review article about tACS pointed out that the frequency of tACS used for attention improvement is generally less consistent [111]. We suggest that future studies should take SHBP or LBP-based single-Hz EEG features into consideration, to establish the frequency parameter for tACS. To be more specific, authors could manipulate those single-Hz EEG features one by one until a significant causal link can be established. Regarding the aspect of practical utility, compared to the aforementioned feedback methods, the following are the hurdles that may impede the use of current-based brain stimulation methods:

(1) The inconvenience of using wet electrodes (usually saline-soaked sponge electrodes) on hairy regions;
(2) The relatively longer stimulation time required before it takes effect (10–30 min).

## 5. Research Challenges

Unresolved problems remain.

### 5.1. Open-Loop Algorithms

#### 5.1.1. The Generalizability

EEG data characteristics vary widely between individuals [62]. Particularly, the drowsiness processes vary from person to person [112]. Therefore, a generalized DDD algorithm that can overcome the inter-individual differences should be developed. Research on generalized features [61] and DM models [62,98] is scarce, and so is the case of generalizability of the DDD algorithms across different ground truths (e.g., two ground truths used in [53]). Johnson’s group committed to detecting drowsiness by using a generalized algorithm [113]. To develop such an algorithm, first, they proposed ensuring maximum stability and inter-individual generalizability by using a large sample size (e.g., 135 participants in their study) and individualizing the model and, secondly, different cognitive tasks should be applicable across each subject.

#### 5.1.2. The Early-Detection

EEG signal is weak and highly vulnerable to motion artifacts, particularly when dry sensors are used. Therefore, developing an algorithm that is robust enough to extract features from motion artifacts contaminated early drowsy symptoms, such as yawning or rubbing the face or eyes, is still a challenging task. Additionally, while the continuous-output-type DM models (e.g., probabilistic models and regression models) are used for increasing the resolution of drowsiness detection and for detecting drowsiness early, their validation is constrained for want of corresponding ground truth. At present, only discrete output-type ground truths are available.

#### 5.1.3. The Practical Utility

Papadelis et al. [114] held the view that, since some modern cars have already been equipped with the built-in eye leads sensors, an EOG-based approach would be more driver friendly and efficient than an EEG-based system and, therefore, should attract greater attention from the industry. Indeed, most of the commercially available DDD systems in the market are video-based [115]. However, the video-based system is not favored much because of the limitations caused by its brightness and face-to-camera distance [34]. Additionally, the recent advances in EEG dry sensors, low-power integrated circuits and wireless communication technologies enable EEG-based DDD transit from research to practical use; for example, an EEG-based commercial solution is already available for the professional drivers of the coal mines in Australia [116]. These drivers can have their brains monitored in the workplace by simply wearing the cap. With the development of wireless and wearable EEG devices, we believe that EEG-based DDD, under naturalistic driving conditions, is a more promising research area.

As regards the comparison of EEG-based DDD solutions with those of the other existing approaches, Sommer et al. [115], using simulated driving experiments, report that the combination of EEG and EOG (EEG/EOG) is better than video-based PERCLOS. Based on a naturalistic driving condition, and comparing other physiological signals, Papadelis et al. [114] concluded that EEG/EOG outperforms ECG and EMG. In contrast to this, Khushaba et al. [24], using a simulated experiment, claimed that EEG/ECG is better than EEG/EOG. They further contend that EEG is the best signal if these physiological signals are used individually. However, the need for comparison of EEG and individual physiological signals, under naturalistic driving conditions, remains unfulfilled.

### 5.2. Closed-Loop Algorithms

The causal link between EEG biomarkers and drivers’ drowsiness levels is the key to a successful closed-loop DDD system. A critical step to verify this causal link is to use neuromodulation approaches, including tDCS and tACS, to directly manipulate those EEG biomarkers found in open-loop approaches. However, apart from prior work from our group [106], we did not find similar studies to rigorously verify the causal relationship between those EEG features and drivers’ drowsiness. Thus, future studies can be planned on this topic, in order to design a reliable closed-loop DDD system.

## 6. Conclusions

For synthesizing the algorithm-level infrastructure, we have organized this article into open-loop studies and closed-loop studies. The open-loop studies are structured into the following three steps: data sensing, data processing and data-to-knowledge. The closed-loop studies are structured in terms of the following two important fields of real-world DDD solution: the methods to enhance attention and the duration of enhanced attention.

On reviewing these techniques, we arrive at the following conclusions:

(1) From the point of view of early detection of drivers’ drowsiness, advanced features, such as HOS (with 30-s time window) and PTFD (with 10-sec time window), are more robust than the traditional EEG frequency bands-based features. In addition, the continuous output-type DM models (e.g., probabilistic models and regression models) outperform the commonly used discrete output-type DM models (e.g., threshold, binary and multi-class classification models).
(2) From the point of view of practical utility, the bipolar single channel in the occipital region is the most suitable EEG montage for DDD research. tDCS technology is most effective in boosting alertness. Its duration of enhanced attention is long-lasting, when compared to that of visual, vibrational and auditory feedback methods.
(3) From the point of view of reliability, PERCLOS+ provides the most reliable ground truth for the development and verification of real-time DDD algorithms.

## Figures and Tables

**Table 1:** Fifty-four Studies on EEG-based DDD listed with montages, as well as time windows for feature extraction, if applied.

Ref.	Number of Channel	Channel Position ^(1)^	Time Window
[31]	33	-	1 min
[35]	19	F1, F2, F7, F8, F3, F4, T3, T4, C3, C4, T5, T6, P3	1 s
[36]	1	O1 or O2	1 s
[37]	1	Fp1	10 s
[38]	4	-	-
[39]	2	C3, P3	10 s
[40]	1	C3	5 s
[41]	1	Fp1 & Fp2	-
[42]	6	Fp1, Fp2, T5, T6, O1, O2	4 s
[43]	1	Oz	8 s
[44]	26	-	5 s
[45]	1	-	2 min
[8]	1	C3 or C4	30 s
[46]	1	C3 & O1	30 s
[47]	2	F7 & T3; F4 & C4	-
[48]	16	-	1 min
[49]	1	O1	1 min
[50]	2	C4, O2	10 s
[51]	2	Fp1 & Fp2; T3 & T4	-
[52]	1	Fp1 & Fp2	2 s
[53]	29	Frontal (F: 3, 1, z, 2, 4; Fc: 3, 1, z, 4), Central (C: 3, 1, 2, 4; Cp: 3, 1, z, 2, 4) and Posterior-occipital (P: 3, 1, z, 2, 4; Po: 3, z, 4; O: 1, z, 2)	20 min
[24]	3	Fz, T8, Oz	1 min
[54]	2	C4, O2	1 min
[55]	19	-	2 s
[56]	1	O1 & O2	1 min
[57]	1	O1 & O2	30 s
[58]	4	Forehand	10 min
[59]	8	Fp1, Fp2, F3, F4, P3, P4, O1, O2	10 s
[60]	1	Fp1	10 s
[61]	1	P3	20 s
[62]	6	Occipital	1 s
[63]	14	F7, F8, T3, T4, T5, T6, F3, F4, C3, C4, P3, P4, O1 and O2	1 s
[64]	2	Fz & Cz; Pz & Oz	-
[65]	21	-	30
[66]	1	C4 & P4	1 min
[67]	4	Occipital	-
[68]	1	Fp1 & Fp2	2 s
[69]	3	Fz, Cz, Oz	2 s
[70]	3	Fz, T8, Oz	10 s
[71]	2	Fz, Oz	1 min
[72]	3	(Fp1, C3, O1) or (Fp2, C4, O2)	30 s
[73]	14	-	1 s
[74]	1	O1 & O2	<1 s
[75]	2	Fz & Cz; Pz & Oz	1 min
[76]	1	O1 & O2	1 min
[77]	4	Occipital	2 s
[78]	2	Fz, Pz	-
[79]	2	Fz, Oz	1 min
[80]	1	-	5 s
[81]	2	Fz & Cz; Pz & Oz	30 s
[82]	19	-	2 s
[83]	1	Fp1	1 s
[84]	1	O1 & O2	30 s
[85]	14	-	-
[86]	18	Posterior-occipital (CP_Z_, CP2, P1, P_Z_, P2, PO3, PO_Z_, PO4, O1, O_Z_, O2) and Temporal (FT7, FT8, T7, T8, TP7, TP8)	8 s

^(1)^ Symbols “&” and “,” relate respectively to bipolar channel (e.g., bipolar single channel: O1 & O2) and unipolar channel (e.g., unipolar two channels: Fp1, Fp2); Symbol “;” is used to separate one bipolar channel from the other (e.g., bipolar two channels: Fz & Cz; Pz & Oz).

**Table 2:** The summary of the time domain features used in EEG-based DDD.

Features		Mathematic Expression
Statistical measure	Maximum (Max) [80]	$\mathrm{Max}=x_{k}\in \left\{x_{1}\ldots x_{N}\right\}$
	Minimum (Min) [80]	$\mathrm{Min}=x_{k}\in \left\{x_{1}\ldots x_{N}\right\}$
	Standard deviation (STD) [76,81,82,83]	$\sigma =\sqrt{\frac{1}{N}\overset{N}{\underset{k=1}{\sum }}{\left(x_{k}-\mu \right)}^{2}}$
	Root mean square (RMS) [70]	$RMS=\sqrt{\frac{\sum_{k=1}^{N}{\left(x_{k}\right)}^{2}}{N}}$
	Integration [42,44,75,80]	$Integration=\overset{N}{\underset{k=1}{\sum }}\left|x_{k}\right|$
The Number of Zero-Crossing (NZC) [42,46,70,80]		$NZC\left(x\right)=\overset{N-1}{\underset{k=1}{\sum }}s\left(x_{k},x_{k+1}\right)$ $s(x_{k},x_{k+1})=\left\{\begin{array}{l} 1\\ 0 \end{array}\right.$ $\left.\begin{array}{c} if\;\left(x_{k}x_{k+1}\right)<0\\ if\;(x_{k}x_{k+1}\geq 0 \end{array}\right\}$
Hjorth parameters [70]		$\mathrm{Activity}:\;Act=\frac{\sum_{k=1}^{N}{\left(x_{k}-\mu \right)}^{2}}{N}$ $\mathrm{Mobility}:\;Mob=\sqrt{\frac{\mathrm{var}({\dot{x}}_{k})}{\mathrm{var}(x_{k})}}$ $\mathrm{Complexity}:\;Com=\frac{Mob\left({\dot{x}}_{k}\right)}{Mob\left(x_{k}\right)}$
Barlow parameters [70]		$\mathrm{Absolute}\;\mathrm{Mean}\;\mathrm{Amplitude}:\;MA=\frac{\sum_{k=1}^{N}\left(\left|x_{k}\right|\right)}{N}$ $\mathrm{Mean}\;\mathrm{Frequency}:\;MF=\frac{\frac{1}{N}\sum_{k=1}^{N}\left|{\dot{x}}_{k}\right|}{MA}$ $\mathrm{Spectral}\;\mathrm{Purity}\;\mathrm{Index}:\;SPI=\frac{\sum_{k=1}^{N}\left|{\dot{x}}_{k}\right|}{\sum_{k=1}^{N}\left|{\ddot{x}}_{k}\right|}$
Auto regressive model coefficients(ARMC) [70]		$x_{k+1}=c+\overset{N}{\underset{k=1}{\sum }}\phi_{k}x_{k}+\varepsilon_{k+1}$ where *c* is the intercept and *φ* is ARMC which specifies how much the *x_k_* contributes to the *x_k_*_+1_. *ε_k_*_+1_ is assumed to be the random zero mean noise.
Entropy	Shannon entropy (SE) [36,45,75]	$SE\left(x\right)=\frac{-\overset{N}{\underset{k=1}{\sum }}p\left(x_{k}\right)\times \log_{b}p\left(x_{k}\right)}{\log_{b}M}$ where *p*(*x_k_*) represents the probability that the *x_k_* occurs anywhere in the EEG samples *x*. The *p*(*x_k_*) is estimated by a histogram method where the *x* is linearly divided into *M* bins.
	R’enyi entropy (RE) [37,45,60]	$H_{\alpha }\left(x\right)=\frac{1}{1-\alpha }\log(\overset{N}{\underset{k=1}{\sum }}p{\left(x_{k}\right)}^{\alpha })$ where *α* is the order, *α* ≥ 0 and *α* ≠ 1
Mean comparison test (MCT) [61]		$M\left(i\right)=\frac{\mu_{1}-\mu_{2}\left(i\right)}{\sqrt{\frac{\sigma_{1}^{2}}{t_{1}}+\frac{\sigma_{2}^{2}\left(i\right)}{t_{2}}}}$ where *μ*_1_ indicates the fixed reference window, *μ*_2_(*i*) indicates the *i*th dynamic window.
Mahalanobis Distance (MD) [43]		$M\left(x\right)=\sqrt{{\left(x-\mu \right)}^{T}S^{-1}\left(x-\mu \right)}$

**Table 3:** The summary of the FFT-based features used in EEG-based DDD.

Features	Mathematic Expression	References
Pure Band Equation (PBE)	*θ*/*β*	[44,50,56]
	*θ*/(*α* + *β*)	[51]
	(*θ* + *α*)/*β*	[44,51]
	(*θ* + *α*)/(*α* + *β*)	[44,51]
	*θ*/*α*	[73,79]
	*δ*/*α*	[73]
	*α*/*β*	[44,58,85]
	(0.6 * *θ* + 0.4 * *α*)/(0.5 * *β*)	[52,69]
	(*α* + *β*)/*δ*	[45]
	(*δ* + *θ*)/(*α* + *β*)	[48]
Relative Band Power (RBP)	$RBP\left(z_{i}\right)=\frac{Power\left(z_{i}\right)}{\sum_{i=1}^{5}Power\left(z_{i}\right)}\times 100\%$, where, *z_i_* = {*δ*, *θ*, *α*, *β*, *γ*}.	[19,61,66,82]
Log Band Power	LogM, where M∈ {8, 9, 10, 11, 12 Hz} or {10, 11, 12, 13, 14 Hz}	[26]
	Log(*α* − *θ)*	[71]

**Table 4:** Ground Truths used in EEG-based DDD.

DM Model	No.	Ground Truth
Threshold/Binary	1	Subjects’ response time to lane departure event [43,67,92]
	2	Subjects’ response time to sound simulation [42]
	3	Subjects’ collision rates with time [58]
	4	Subjects’ self-assessment [52,68] (Subjects press a button, placed next to them, when feeling drowsy)
	5	Subjects’ self-assessment [69] (Alert: KSS < 8 and KDS = 0; Fatigue: KSS ≥ 8 and KDS ≥ 50)
	6	Subjects’ self-assessment [53] (Alert: KSS < 8.5; Drowsy: KSS ≥ 8.5)
	7	Subjects’ self-assessment [42] (Alert: KSS < 7; Drowsy: KSS ≥ 7)
	8	Subjects abort driving due to severe fatigue [53,85]
	9	RK (Wake, Stage I) [46,66,76,80]
	10	Facial features that are manually identified by video recording [56] (Drowsiness: Wierewille scale ≥ 3)
	11	Authors’ self-assessment, based on the subjects’ response during the experiment (The subjects need to accurately count the number of a visual stimulus shown [36])
	12	Authors’ self-assessment, based on the experimental video recording and the subjects’ self-assessment [44]
	13	Authors’ self-assessment, based on subjects’ eye and head movements [35]
	14	Assessment of Driver’s Vigilance and Warning according to Traffic Risk Estimation (AWAKE): Index ≥ 1 represents drowsiness [61]
	15	PERCLOS [76]
Multi-class	16	Subjects’ self-assessment (ESS) [55,59] (Alert: ESS < 8; Drowsy: 8 ≤ ESS ≤ 11; Severe drowsy: ESS ≥ 24)
	17	Facial features that are manually identified by video recording (Wierewille scale) [24,70,79]
	18	Self-assessment (KSS) [71]
	19	RK (Wake, Stage I, Stage II) [72]
	20	Authors’ self-assessment, based on their own experience [73]
	21	Unknown sleep scoring standard [40]
Regression	1	Subjects’ response time to lane departure event [62,77]
	17	Facial features that are manually identified by video recording [50] (Wierewille scale)
	22	Subjects’ driving error index [31]
Probabilistic	15	PERCLOS [86,87]
	23	Self-assessment [56,84] (Subjects press buttons on the steering wheel when feeling arousal, a little bit drowsy and drowsy)
Transfer	1	Subjects’ response time to lane departure event [98]

**Table 5:** EEG-based DDD Accuracies obtained by using Pure Threshold-based Models and Various Features and Ground Truths.

Ref. No.	Features	Acc (%)	Sens (%)	Spec (%)	GND Truth No.
[54]	FFT+: a wide range of *α* band	-	74.4	95.5	10
[52]	BPE: #8	90.4	-	-	4
[49]	PBP: *θ~β* bands	83.8	-	-	-
[43]	FFT+: MDT and MDA	82.8	-	-	1
[83]	FFT+: mean and STD extracted from *α* and *β* bands	81	-	-	9

**Table 6:** EEG-based DDD Accuracies obtained by using Binary Classification Models and Various Features and Ground Truths.

Ref. No.	Features	Models	Acc (%)	Sens (%)	Spec (%)	GND Truth No.
[72]	SHBP (1~27 Hz) and BPE: #5	RBF-SVM	97.48	-	-	9
[76]	RPB: *α* band	Linear-SVM	95.22	100	93.8	15
[85]	Wavelet: WPT features that are selected by CSP method	SVM	94.2	-	-	8
[44]	BPE: #1, 3, 4, 7 and PBP: *δ~β* selected by PCA and fish score	SVM	92.2	-	-	12
[42]	Wavelet+: NZC and IEEG extracted from *θ~β* bands	ANN	-	90.91	79.1	2
[77]	FFT+: IEEG, SE and STD extracted from *δ~γ* bands	SVM	92.5	85	100	9
[69]	FFT+: DF, APDP, CGF, FV and MPF extracted from *δ~β* bands	RBF-SVM	75	86	64	5
[61]	FFT+: RBP-based MCT values	FI	-	84.6	82.1	19
[80]	Hybrid: three features from time-domain (Max, Min, STD); ten features from FFT-based methods (CenF, PF, RH/L, Q1F, Q3F, spectral STD, IR, MF, AC and KC); Wavelet-based methods (IEEG and NZC from *θ~β* bands)	LDA-ANN	-	83.6	87.4	9
[35]	PBP: *δ~β* bands	ANN	81.49	80.53	82.44	13
[36]	FFT+: SE extracted from SSVEP-based power spectrum	Single-layer feed-forward ANN	72.5	-	-	11

**Table 7:** EEG-based DDD Accuracies obtained by using Multi-class Classification Models and Various Features and Ground Truths.

Ref. No.	Features	Models	Acc (%)	GND Truth No.
[24]	Wavelet+: Normalized log energy of the wavelet-packet coefficients that are selected by FMI method	LDA	97% (5 levels)	17
[39]	Wavelet: band power	Multilayer perceptron ANN	95~96% (3 levels)	21
[70]	Hybrid features: TDAR, selected by FNPA	RBF-SVM	93% (5 levels)	17
[64]	Wavelet+: Normalized log energy of the wavelet-packet coefficients selected by FMI method	SVM	91% (5 levels)	1
[73]	Wavelet+: FFT band power and SC generated by WPT	Subtractive FI	84.41% (4 levels)	21

**Table 8:** EEG-based DDD Accuracies obtained by using Regression Models and Various Features and Ground Truths.

Ref. No.	Features	Models	Acc (%)	GND Truth No.
[77]	LBP: Log-transformed SHBP (1–30 Hz)	RBF-SVR	*r^2^* = 0.932 and RMSE = 0.124 (s)	1
[31]	Wavelet+: Normalized log energy of the wavelet-packet coefficients, selected by FMI method	MLR	*r^2^* = 0.778	22
[62]	PBP: *θ* and *α* power	SONFIN	*r^2^* = 0.613 and RMSE = 0.360 (s)	1
[50]	FFT+: *α* burst duration, mean amplitude, relative amplitude, amplitude variance, wave duration variance, wave similarity, slope smoothness measurement	MLP	*r^2^* = 0.272	17

**Table 9:** Performance Comparison of Approaches for Vigilance Enhancement.

Ref.	Core Approach	Max Duration of Enhanced Vigilance Level	Technical Parameters	Intervening at Slightly Drowsiness Moment	Including Neurofeedback
[67]	Auditory	40 s	EEG-guided 1750 Hz tone per sec	No	Yes
[103]	Caffeine	2 h	-	No	No
[103]	tDCS	6 h	Hairy area; 30 min and fixed 2 mA session	No	No
[105]	tDCS	-	Hairy area; 20 min and fixed 1.5 mA session	No	No
[106]	tDCS	23 m	Non-hairy area; EEG-guided stimulation duration and fixed 2 mA	Yes	Yes

## Data Availability

This is a review article, no new data generated. All papers reviewed in this article can be downloadable from public paper database. More details can be found in the section References.
